# Progress towards Sustainable Utilisation and Management of Food Wastes in the Global Economy

**DOI:** 10.1155/2016/3563478

**Published:** 2016-10-26

**Authors:** Purabi R. Ghosh, Derek Fawcett, Shashi B. Sharma, Gerrard Eddy Jai Poinern

**Affiliations:** ^1^Murdoch Applied Nanotechnology Research Group, Department of Physics, Energy Studies and Nanotechnology, School of Engineering and Energy, Murdoch University, Murdoch, WA 6150, Australia; ^2^Department of Agriculture and Food, 3 Baron-Hay Court, South Perth, WA 6151, Australia

## Abstract

In recent years, the problem of food waste has attracted considerable interest from food producers, processors, retailers, and consumers alike. Food waste is considered not only a sustainability problem related to food security, but also an economic problem since it directly impacts the profitability of the whole food supply chain. In developed countries, consumers are one of the main contributors to food waste and ultimately pay for all wastes produced throughout the food supply chain. To secure food and reduce food waste, it is essential to have a comprehensive understanding of the various sources of food wastes throughout the food supply chain. The present review examines various reports currently in the literature and quantifies waste levels and examines the trends in wastage for various food sectors such as fruit and vegetable, fisheries, meat and poultry, grain, milk, and dairy. Factors contributing to food waste, effective cost/benefit food waste utilisation methods, sustainability and environment considerations, and public acceptance are identified as hurdles in preventing large-scale food waste processing. Thus, we highlight the need for further research to identify and report food waste so that government regulators and food supply chain stakeholders can actively develop effective waste utilisation practices.

## 1. Introduction

Food is a basic human need, while food waste has been identified as a major challenge facing humanity today [1]. Currently, around 21,000 people die every day due to hunger related causes [2] and globally one in nine people go to bed each night hungry [3]. Nevertheless, approximately one-third of all food produced today goes to landfill [4]. The vast amount of food ending up as waste is not only a humanitarian problem, but also a serious economic and environmental problem [5–7]. The world has limited natural resources and environmental benign cost-effective solutions must be found to increase food production, improve distribution networks, and promote effective food supply chain management practices [8]. To alleviate the increasing demand for food production, it is necessary to significantly reduce food waste. Reducing food waste is an important factor that can significantly improve the overall efficiency of the food supply chain [6]. Researchers in the field maintain that sustainable food production, intelligent management, and proper food distribution are the key factors that must be addressed if we expect to feed the predicted 12.3 billion people in 2100 [7, 9]. So, reducing food waste becomes a priority, since waste will continue to be generated throughout the food supply chain if no action is taken. Companies involved in the food supply chain and the population at large will continue to waste food as long as they can afford to waste. Importantly, food waste results in loss of time, effort, and the other resources that went into producing that food. Other resources lost include fertilizers, pesticides, and the soil and water. From an environmental perspective, food lost or discarded each year accounts for 3.3 billion tonnes of carbon dioxide emissions globally. The scale of food waste globally can be quite staggering and several significant examples are presented in Table 1 so that the reader can appreciate the magnitude of the problem. Thus, governments, industry, and communities must work collaboratively to achieve policy and cultural change towards prevention of food waste at all levels [10, 11]. Therefore, to keep pace with the ever increasing demand for food, it is essential to adopt a policy that says “no” to food waste.

Defining food waste is not always straightforward since distinguishing between edible and nonedible parts of food is subjective. In some parts of the world, a food judged edible may be considered nonedible in other parts. Naturally, not every part of an agricultural or livestock product is entirely edible and there will always be unavoidable nonedible parts such as citrus fruit zest, fruit stones, bones, and eggshells [12, 13]. In many cases, the difference between edible and nonedible is not clearly defined and depends on dietary habits (consumption of bread crusts, apple or potato peel, fat on meat, etc.), food culture, and geographic location. In the present study, food that is not consumed by the end user, which includes the nonedible parts of the food, is considered to be “food waste.” All food products go through a life cycle, starting from the farm and progressing through processing, distribution, retail, and finally consumption and/or dumping as presented in Table 2. Inspecting Table 2 reveals that food waste occurs throughout the entire food supply chain. The degree of food waste depends on factors such as (1) developed and developing country [6, 14]; (2) prevailing weather conditions and pest management protocols [15]; (3) storage, transport facilities, and processing efficiency [16–18]; (4) market demand and visual appearance of produce [14]; (5) consumer acceptance of produce [19] and consumer affordability to waste [4].

Even a couple of decades ago, food waste was not considered to be a significant economic cost or a waste of natural resources [20]. However, growing public concerns about hunger, conserving the environment, and the effect of socioeconomic factors have accelerated research into food waste. Food waste research is aimed at finding better ways of using this natural and renewable resource [17]. Unfortunately, there will always be a certain amount of waste produced in the food supply chain. However, current levels of waste occurring in the food supply chain are much greater than other industries and arise from the lack of willingness or inability to coordinate the various activities along the chain [21–23]. Therefore, to make the food supply chain more sustainable and effectively manage food waste, a much deeper understanding of the current state of affairs is needed [24]. This not only means food waste itself, but also means taking into account associated factors like greenhouse gas emissions (GHG) and the use of other resources such as water, land, labour, money, and energy. After taking all these factors into consideration, it is also very important to make the various stages in the food supply chain such as production, distribution, and marketing more efficient and sustainable [25].

Generally speaking, the literature in the field often reports the importance of effective food waste management to reduce problems such as large waste volumes going to landfill, landfill gas emissions, landfill leakage contaminating waterways, and costs associated with transport and handling of wastes. Alternatively, many food wastes can be considered as a valuable source of nutrients with the potential to be processed into products to feed the world's increasing population [14, 26]. Recently, Mirabella et al. reported a range of nutrients available from fruit, vegetable, dairy, and meat and fish wastes that could be used in food products (gelling agent in confectionary, fat replacement in meat products, supplementary food products, and seafood flavours for soups) and beverage preservatives [1]. Food wastes have also been considered as a source of renewable energy with the potential to significantly reduce the current dependency on energy derived from fossil fuels [27, 28]. Using food waste as an alternative energy source has the advantage of reducing the amount of waste going to landfill and diminishing the associated problems of gas emissions and groundwater contamination [29, 30]. The use of food waste also alleviates the problem of land competition between food crops and crops for liquid biofuels [31].

The present review provides an overview of current research into terrestrial and aquatic food waste and progress towards utilising the waste. The review examines the various causes that result in food waste and also presents information regarding waste levels throughout the different stages in food supply chains operating in several regions around the world. Also discussed are the socioeconomic aspects of food waste, the willingness to implement food waste initiatives that promote efficient and sustainable food chain management practices. In addition, probable future trends and initiatives for the implementation of effective ecofriendly and sustainable approaches for managing food wastes are outlined.

## 2. Terrestrial Food Waste in the Food Supply Chain and Current Waste Utilisation

### 2.1. Crop Waste in the Food Supply Chain and Current Waste Utilisation

Crop waste begins at the farm and continues throughout the food supply chain. Between farm and fork, food waste is produced by each of the six stages of the food chain as detailed in Table 2. In developed countries, food waste can be quite significant even at the agricultural or harvest stage. Food waste can result from factors such as produce sizing and aesthetic standards, produce quality regulations, production surpluses, and economic factors. For example, in 2009, Italian agricultural produce estimated to be 17.7 million tonnes was left in the ground and equated to around 3.25% of total produce production [32]. Surprisingly, some studies have indicated that the agrofood sector waste could be as large as 40% of the total production value [33], while studies in the Netherlands have revealed that annual food wastage costs are around € 4.4 billion (US$ 4.9 billion). End-consumers waste around €2.4 billion (US$ 2.7 billion) or about 10% of all food purchased and the remaining €2 billion (US$ 2.21 billion) was wasted through the various stages of the food supply chain [33, 34].

In a Swedish study, 16 different horticultural products including typical fruits and vegetables sold by retailers were responsible for wastes ranging from 0.4% to 6.3% of produce [35]. Similar studies have also found that fruits and vegetables are the main source of household food waste and equate to around one-third of purchased food products [36].

For instance, in the United Kingdom (UK), potatoes came first in a ranking of 100 fruits and vegetables and accounted for around 0.4 million tonnes (10%) of total waste produced annually [37]. Australians, for example, throw away around AU$ 1.1 billion (US$ 0.84 billion) worth of fruits and vegetables each year making them the largest food waste category [19]. Studies have shown that fruits and vegetables are the most wasted food category among all terrestrial and aquatic food products in both developed and developing countries as seen in Table 3. Moisture content, temperature sensitivity, and delicate surface membranes make fruits and vegetables susceptible to spoilage during production, transportation, and storage. This susceptibility often leads to large amounts of waste throughout the food supply chain. For example, in Switzerland, around 47% of all vegetables produced are wasted in the food supply chain. And in Germany fruits and vegetables account for 43% of all household waste as seen in Table 3.

In many cases, the results of these studies are not comparable, since they did not assess the whole food supply chain (only looked at specific stages and waste types) and were carried out by different researchers worldwide using different assessment protocols. For example, a number studies on fruit and vegetable waste fail to take into account grains and root/tuber wastes. And others have taken into account wastes generated from grains and root/tubers in an attempt to minimise and simplify data collection. Many consumer and retail waste assessments contain very little information about farm practices, processing waste, and wastes resulting from storage and transportation. In spite of their importance, consumers and retailers cannot be considered as the only contributors to waste in the food supply chain. Nevertheless, it is extremely difficult to obtain detailed information from all stakeholders involved in the food supply chain because of business confidentiality considerations. Another limitation arises from the types of measurement procedures used to record and analyse food waste data around the world. In addition, making comparisons is difficult because waste levels can be presented in terms of percentage waste, local currency, and even weight loss. Furthermore, variations can occur between different regions within a country where economic, social, and behavioural reasons may promote specific types of food wastage.

Determining waste levels in a food supply chain often reveals that they are high and costly. For example, in 2008, the United States of America (USA) produced three large waste streams consisting of grain (US$ 34,791 million), vegetables (US$ 103,417 million), and fruits (US$ 62,146 million) at considerable economic cost [13]. Furthermore, each year, the USA produces more than 2.7 million tonnes of fruit and vegetables that are not harvested or remain unsold due to poor crop aesthetics and low market prices [37]. Moreover, most studies only measure or estimate a particular food waste and fail to address any trends in the levels of wastage. To remedy this situation, the United states Department of Agriculture (USDA) carried out a detailed analysis to understand the variation in food waste levels between 1995 and 2010. This data is presented in Table 4 and shows a downward drift in consumer waste compared to retailers waste, although consumers are often blamed for high waste levels. Also, over this period, retail waste has increased for most commodities especially grain products, while consumer waste levels have significantly decreased for some food products such as grain products and fruits. In particular, vegetable waste produced by consumers in 2008 and 2010 was significantly lower than waste levels recorded in 1995.

For developing countries, around 15 to 50% of all fruit and vegetable waste occurs in the postharvest stage [38–40]. For example, in Africa, cassava wastes can be as large as 45% [41] and yam waste levels can reach 50% [42]. In the Philippines, fruit wastes from crops such as papaya can range between 30 and 60% of the total crop [12]. Similarly, around 18 to 40% of all fresh fruits and vegetables go to waste in India every year due to the lack of refrigerated transport and high quality cold storage facilities. This equates to an annual cost to food manufacturers and sellers of around US$ 71,481 million [43]. Unfortunately, much of this data comes from studies carried out almost 40 years ago and because no recent studies have been carried out there is no current assessment of crop waste levels. Thus, there is a critical need for follow-up studies that take into account factors such as technological innovation, population growth, and consumer and marketing trends. It is critical for researchers to document current food waste levels so that stakeholders such as growers, processors, transporters, retailers, and consumers can take steps to address this growing global problem.

**Figure 1:**
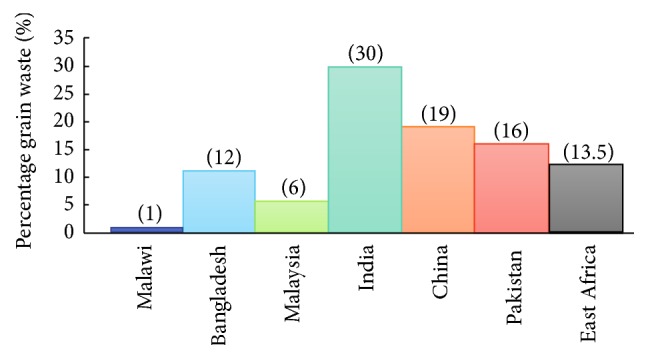
Percentage grain waste in selected developing and less developed countries.

The second major food group after fruit and vegetables is grain. Among the grains, rice is recognised as the world's second staple food and on average has a waste level equivalent to 15% of total global production [44, 45]. However, this waste level is not the same for all countries due to variations in climatic zones and various production practices in each respective country. In particular, storage practices in both developing and less developed countries have been extensively studied and reveal significant differences between countries. For example, grain storage waste levels range from less than 1% in Malawi [46, 47] to 12 to 13% in Bangladesh and 3 to 6% in Malaysia [48] as seen in Figure 1. Studies have also shown that grain waste in the Chinese supply chain is 19.0%  ± 5.8%, with consumers accounting for the single largest portion of the waste (7.3%  ± 4.8%) [49]. And postharvest and preprocessing cereal waste in sub-Saharan Africa was estimated to be around US$ 4 billion. This extremely large cost equates to 13.5% of the total cereal production produced by countries in this region [50].

Inadequate storage capacity, poorly distributed warehouses, lack of adequately designed storage facilities, and inefficient transport and handling management lead to waste levels of around 20 to 30% of India's total grain production [51]. This level of grain waste is estimated to cost around US$ 14 billion each year and is the highest in the region as seen in Figure 1. Amazingly, this level of waste has the potential to provide the minimum annual food requirements of at least 48 million people in India [52]. In Pakistan, grain waste accounts for around 16% of total production, or 3.2 million tonnes annually, and results from inadequate storage infrastructure that permits widespread rodent infestation [53]. Current data indicates that global postharvest crop wastes have direct consequences in terms of food security, malnutrition, and poverty. Except for Malawi, an African country reporting low grain waste levels, other eastern and southern regional African countries have reported waste levels equivalent to around US$ 11 billion or 13.5% of total grain production. Unfortunately, there is very little information available reporting grain wastes in central or West African regions [54]. Most grain waste reports do record total percentage waste for each country but do not give individual crop wastes such as maize, rice, wheat, and barley. Because these reports do not provide individual information on specific grain crops, it is difficult to determine which are more prone to waste. In spite of this, it is evident that policy, political management, natural calamities, storage, infrastructure facilities, and transportation are the main drivers for producing grain waste in developing countries [55].

**Figure 2:**
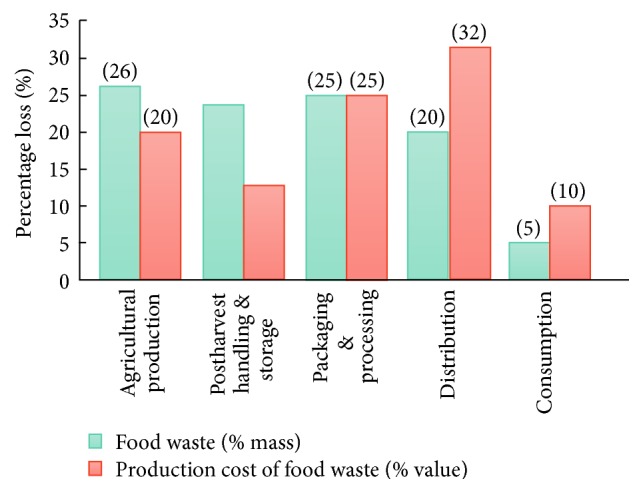
Comparison between food waste quantities (%, by mass) and the cost of food waste (%, by value) in each stage of the food supply chain in South Africa [62].

Food waste not only costs money, but also consumes other resources such as land, water, energy, and labour. When it comes to water usage, South Africa's (SA) food waste costs become significant since it is one of the world' driest countries. For example, approximately 30% of SA's crop production depends directly on irrigated water, while fruit and vegetable production consumes around 90% of all irrigated water used [56]. The total cost of food waste in SA each year is estimated to be around US$ 5.27 billion and equates to 2.1% of the country's gross domestic product (GDP). Furthermore, agricultural production is more prone to waste than processing, packaging, and consumers.  Figure 2 presents a comparison between food waste quantities and the food waste costs for each stage in the SA food supply chain. Interestingly, packaging and processing have similar waste levels and production costs. This suggests that both stages are not cost-effective and rather prone to wastage. While consumer waste levels are relatively low, distribution and infrastructure waste levels are relatively high. The results of this study clearly indicate the severity of waste levels within the SA food supply chain [57].

Grain waste studies have mainly focused on developing countries, with very few studies reporting grain waste in developed countries. A small number of studies conducted in the USA have only investigated grain waste in the retail and consumer stages of the food supply chain as presented in Table 5 [13, 58–60]. It should be noted that in most developed countries grains are considered as livestock feed rather than human food [61]. Thus, there is a crucial need to undertake grain waste studies in developed countries and determine the alternative utilisation of grain and grain wastes as livestock feed. Thus, the lack of reliable food waste data from around the world and the increasing importance of food security mean that significant efforts are needed to fill in the knowledge gaps.

Some studies have just recorded food waste levels, while others have also highlighted methods for managing wastes. There are numerous reports in the literature discussing various recycling and utilisation methods available for processing fruit, vegetable, and grain wastes. The aim of food waste utilisation is to extract the maximum practical benefits and reduce the amount of waste going to landfill [63]. Although there has been extensive information discussing various waste recycling strategies for dealing with agricultural waste, there is very little information assessing the economic benefits of the various waste utilisation methods. At present, there are very few reports available discussing the utilisation of agrowastes on a commercial scale and methods to overcome barriers that currently prevent effective food waste management strategies. All food wastes are a rich source of natural biomolecules and compounds. Fruit and vegetable wastes including peels, stones, and fibres contain a wide range of natural compounds, while grain wastes derived from straw, bagasse, cobs, cotton husk, groundnut husks, and fibrous remnants of forage grasses are mainly composed of useful materials such as cellulose, hemicelluloses, and lignin [64]. Arguably, grain wastes are the most abundant agricultural wastes and the most underutilised [65]. On the whole crop wastes are a valuable source of useful compounds, chemicals, and pharmaceuticals [66]. And currently there is a high demand for pharmaceutical ingredients such as enzymes, solvents, and surfactants all of which can be derived from crop wastes [67]. Because of the rich source of natural compounds found in crop wastes, the European Union, USA, Canada, Japan, and Malaysia are ambitiously developing and promoting an ecofriendly biologically based market. For example, in 2010, the USA placed a replacement target of 12% on all of its chemical feedstock and by 2030 it is expected that bio-based products will have a market share of around 25% [68]. At present, only a small number of bio-based compounds derived from crop sources have made it into commercial products. Typical examples include (1) succinic acid from crops like sugarcane, maize, rice, barley, and potato [68]; (2) starch based plastic production from cassava, maize, and wheat [69]; (3) surfactants from tropical oil producing grains [70]; (4) fatty acids from coconut and oil palm [71]; (5) polymers, lubricants, adhesives, solvents, and surfactants from rapeseed and sunflower [72]; and (6) lactic acid from carbohydrate containing crops such as cereals, potato, and sugar beet [67]. However, to date, very few products containing compounds and chemicals derived from crop wastes have made it into the commercial marketplace. Estimates of market size, market price, potential bio-based share, potential bio-based production size, potential impact for local producers, potential local employment, and prospects for development are very low and rather poor [73]. Therefore, before large-scale development of bio-based renewable products can take place, more detailed feasibility studies and practical business models are needed. Thus, long-term collaborations between producers, manufacturers, and business are needed to undertake further translational research to bring these new and novel products to the marketplace.

Present research has also shown that grain wastes can be used as a source of bioenergy in the forms of bioethanol, biodiesel, and biogas [74]. For example, bioethanol is currently produced from corn in the USA, European Union, and China [75]. In tropical regions such as in Brazil and Columbia, bioethanol is mainly produced from sugarcane [76]. Unfortunately, because of the constraints imposed by available arable land, there is competition between crops specifically grown for biofuel and those grown for food and feed production [77]. Because of this competition, it is not feasible to increase biofuel production using currently available land and technologies. Consequently, current research has focused on developing more advanced or 2nd-generation biofuel production technologies that use wastes derived from grains, fruits, and vegetables. In the last decade, significant progress has been made in developing chemical processes that can convert agrowastes into ethanol. However, major barriers such as the high cost of pretreatments and inefficient conversion processes have prevented the commercialization of large-scale bioethanol processing facilities [78–80]. Further economic analysis has also identified costs barriers such as feedstock chemicals and capital investment that includes pretreatment facilities, fermenters, and steam generation systems as the main factors restricting large-scale processing facilities [81]. Therefore, to overcome many of these barriers, further research is needed to improve efficiencies in current plant and equipment and to explore and develop new agrowaste conversion technologies.

Research into the generation of biogas from fruit and vegetable wastes has also been carried out. But large-scale commercially viable biogas production is still in its infancy. Currently, municipal wastes are recycled through anaerobic digesters to produce biogas, but agrowastes are yet to be converted using this type of processing facility. The main reasons for this are (1) providing a continuous supply of agrowastes to the facility and (2) developing cost-effective transportation between waste sources and facilities. Thus, without a continuous supply of feedstock, the facility is unable to efficiently deliver a steady flow of biogas. Therefore, the continuous supply of agrowaste essentially becomes a Vehicle Routing Problem (VRP) [82]. VRP is one of the most comprehensively studied problems in transportation literature. However, VRP has not been specifically applied to transporting food wastes produced by a food supply chain. Instead, some studies have considered transporting large amounts of food wastes directly between supply points and processing facilities [83–86]. In the case of crop wastes, they are produced at farms, processing facilities, wholesalers, and retailers and are typically spread over fairly large regional areas including both urban and rural ones. Therefore, there is a need to collect wastes from various dispersed locations and transport them to processing facilities. Thus, collecting and transporting food wastes are fundamentally different from harvesting and shipping agriculture crops to market. The difference arises from waste delivery trucks not receiving a full load at any one of the locations. For example, a business may only produce a small amount of food waste that does not make a full load. This necessitates the truck to make multiple pickups from other locations before making its delivery to the processing facility. This type of truck routing is a major cost to food waste collection that has not been fully investigated and could limit large-scale crop waste utilisation. Importantly, while the impact of large-scale biogas operations using first- or second-generation biofuels is being debated, there is also considerable interest in developing small-scale biomass processing to produce biofuels. The advantage of small-scale biofuel production plant is that it enables local communities to access a renewable energy source. Small-scale biofuel plants can utilise locally produced food waste and reduce the dependence on fossil fuels and wood resources.

### 2.2. Livestock, Poultry Meat, and Egg Waste in the Food Supply Chain and Level of Utilisation

Livestock and poultry waste occurs in the early stages of production with animal deaths and animals unsuitable for slaughtering [87]. In the meat industry, the majority of the waste is produced during slaughtering and consists of various nonedible parts that are categorised as byproducts [87, 88]. Meat byproducts consist of bones, tendons, skin, and contents of the gastrointestinal tract, blood, and internal organs. However, these waste parts can vary between each type of animal [89]. Generally, meat products have a relatively short shelf-life ranging between 7 and 26 days [18]. It is for this reason that meat products immediately go to waste if not sold within the labelled expiry date and this is the main reason for wastage at the retail stage. Other reasons for meat product waste include packaging size and date confusion among consumers as detailed in Table 6. Buzby and Hyman in 2012 [13] estimated the total value of meat product waste in the USA at US$ 83,127 million. Their study found that consumers and retailers were responsible for around 35% and 5%, respectively, of the total waste produced, while the total value of poultry waste was estimated at US$ 69,100 million, with consumers being responsible for 37% of the total waste [18]. Studies have also revealed a positive growth trend in meat and poultry waste as presented in Table 4. Overall, there has been an increasing trend in meat consumption around the world with the USA recording the largest increase [90]. The increased consumption is around three times as large as the global average; however, at the same time, trends in retail and consumer waste levels are not clearly understood as seen in Table 4 [91]. Furthermore, a study carried out in Canada analysing food waste data between 1961 and 2009 found that red meat accounted for 39.73% of the total waste and poultry waste was estimated to be around 40.74% [92]. However, this analysis did not include bone waste in the slaughterhouse since no data exists. Moreover, it is crucial to note that these percentage wastes only reflect wastage at the consumption stage in the food supply chain and do not take into account farming, processing, and distribution waste data. Similarly, Australian consumers waste around AUS$ 872.5 million (US$ 637.5 million) worth of meat and fish every year [19]. Unfortunately, meat and poultry waste has not been studied to the same extent as fruit and vegetable wastes. However, a limited number of studies presented in Table 6 do indicate that most of the waste is produced in the processing of livestock and poultry and the trend is steadily increasing as indicated in Table 4. Inspection of Table 6 reveals that meat processing in the UK accounts for 56% of all wastes [36, 93] and in the grouping of 14 European countries processing wastes vary between 35 and 42% [94]. Unfortunately, there is no data available recording the amount of waste generated during the preslaughtering stage of meat and poultry production.

Like fruit and vegetable wastes, meat and poultry byproducts are also rich in nutritional, medicinal, and pharmaceutical materials [95]. The broad diversity of products has the potential to be used in human food products, animal feeds, fertilizers, and biofuels [96]. Currently, both academic and industry researchers are investigating various methods of adding value to meat and poultry products and make better use of their byproducts. Current research, using the most up-to-date analysis techniques, has been aimed at determining nutritional properties, bioactive molecules, and other useful chemical compounds commonly found in these byproducts. Many of these bioactive molecules and chemical compounds have the potential to be used in fields such as cosmetics and pharmaceuticals [97]. In many countries, slaughterhouse wastes have already been used to produce cattle and poultry feed, since these wastes are an excellent source of many different types of proteins [95]. Two animal byproducts that have been used without further processing by the fast food industry are tallow and lard. Unfortunately, consumer anxiety in recent years has restricted the use of these byproducts in the fast food industry [98]. In many cases, meat, poultry, and dairy processing wastes have the potential to be recycled and processed into higher value and useful products. But inappropriate use of recycled meat byproducts can create major aesthetic and even health problems. Therefore, most countries have regulatory requirements that limit the use of meat and poultry wastes in the interests of food safety and quality. Also, economic factors have limited the viable use of meat and poultry wastes. For example, at one time, Japanese meat and poultry wastes were extensively used in animal feed until a relatively low priced imported feed concentrate entered the marketplace. And as a result waste usage declined and out of the 20 million tonnes of wastes being produced each year only 3% was used as fertilizer and 5% as animal feed [99]. The remaining large amounts of waste were incinerated or ended up in landfills. In an attempt to reduce the number of enormous landfill sites, reduce the environmental burden, and prevent gas emissions, the Japanese government introduced a new food-recycling law in May 2001. Unfortunately, just after its introduction, an outbreak of Bovine Spongiform Encephalopathy was reported and created a very negative public response to food recycling. Consequently, public concerns and safety issues have prevented food recycling for human and ruminant consumption [99]. The only other way of processing meat and poultry waste in Japan is via compost production, but to date there has been limited acceptance of this product by farmers.

Eggs are an important food and are extensively used in cooking for the production of a diverse range of food products. In Korea, the annual consumption of eggs was estimated to be around 540,542 tonnes and is expected to increase every year [100]. Because of the extremely large amounts of eggs used worldwide, there are also large amounts of egg wastes produced. For instance, in Switzerland, 18% of all egg wastes occur during production, around 9% occurs in the retail sector, and a massive 64% is produced by consumers as seen in Table 7 [8]. In the North American supply chain, 31.4% of all eggs produced end up as waste [59]. And in the USA around 9% of all egg wastes are produced in the retail sector and consumers produce 14% as seen in Table 7 [13]. Importantly, waste products from both poultry processing and egg production industries must be efficiently dealt with, since growth in both industries largely depends on effective waste management [88]. In the case of egg production, eggs are vulnerable to bacterial attack if the outer shells are not properly and quickly cleaned to remove faecal particles which contain various microorganisms [101, 102]. In addition, egg waste can also occur during transportation, distribution, and storage if appropriate supportive environment is not supplied. Furthermore, because of the extremely large numbers of eggs used worldwide, approximately 50,000 tonnes of eggshells is produced each year [102]. These eggshells contain high levels of calcium carbonate (CaCO_3_) that could be used as an alkaline compound to immobilise heavy metals. Therefore, recycling eggshells for the immobilization of heavy metals in wastewater has the potential to significantly reduce environmental pollution [103]. Accordingly, there have been a number studies evaluating eggshells as immobilising agents for heavy metals such as chromium(III) and lead [104–106]. However, to date, the practical use of waste eggshells as immobilising agents is still largely unknown [95].

### 2.3. Dairy Waste in the Food Supply Chain

The dairy industry, because of its worldwide importance, has been extensively studied to determine its environmental impact. The most important product produced by the dairy industry is raw milk. Raw milk is processed into products such as consumer milk, butter, cheese, yogurt, condensed milk, dried milk (milk powder), and ice cream [107]. In spite of being extensively studied, what is lacking is a comprehensive understanding of waste levels produced throughout the whole dairy industry. The agricultural stage is often reported as the main source of wastes in the life cycle of milk and dairy products [108–110]. However, studies in the UK and Spain have identified the main causes of milk waste coming from poor product quality during the summer period, poor forecasting, packaging mistakes, and breakages occurring at the retail stage [18]. The study also found that poor sales forecasting, slow sales, and cold storage problems during transportation also contributed to the wastage of many dairy products. In addition, cleaning and packaging processes associated with dairy products were also found to significantly contribute to waste levels [111]. Furthermore, dairy product packaging has also been found to significantly contribute to environmental degradation [112]. Generally, waste levels in the dairy industry are quite high. For example, the Mexican milk industry generates between 3.74 and 11.22 million m^3^ of waste products each year, which equates to around one to three times the volume of milk produced annually [113]. And, in the case of Denmark, milk and dairy products contribute around 71,000 tonnes of food waste annually [87], while in the grouping of 14 European countries around 43 to 48% of milk and dairy wastes were produced in the processing stage [94]. In North America supply chain wastage was found to be around 32% [59] and USA retailers were found to waste around 9% of all dairy products as seen in Table 7 [13].

Other sources of milk and dairy produce waste result from frequent product changes, but this can be reduced through appropriate product sequencing and more efficient product scheduling [114]. Other methods of reducing milk wastes include capture of fat, protein, and sugars from wastewater produced during milk processing using processes such as evaporation, centrifugation, ultrafiltration, reverse osmosis, and bioconversion. These recapture processes can significantly reduce the amount of milk and dairy wastes being discharged into the environment [5]. Waste reduction can also have a significant impact on product processing efficiency and improved financial returns. For instance, cheese is derived from milk and is widely used as a standalone product and component of many food products around the world. During cheese manufacture, acidified milk is mixed with an enzyme to form solid cheese or casein and the remaining liquid is called whey [115]. The waste whey can have a negative impact if dumped directly into the environment [5]. Today, around 50% of the world's whey production is treated and transformed into various food products. This cost-effective solution adds value to the whey and reduces wastes [116]. Currently, there is a large body of research in the literature that stresses the importance of reducing milk processing waste and wastewater discharge into the environment. However, the amount of milk and dairy wastes being generated throughout the global food supply chain is still largely unknown. Therefore, there is a current need to undertake studies that can identify and document the magnitude of milk and dairy waste occurring throughout the global food supply chain so that proper waste remediation and management steps can be implemented.

## 3. Aquatic Food Waste in the Food Supply Chain and Level of Waste Utilisation

### 3.1. Fish Waste in the Food Supply Chain

Historically, fish has always been an import food source and even today it is one of the most traded commodities in international markets. It was estimated in 2010 that globally around 54.8 million people were engaged in aquaculture and the wider fishing industry [117, 118]. Currently, fish contributes around 16.6% to the total animal protein supply and 6.5% of all proteins consumed by humans worldwide [118]. Fish is highly regarded for its carbohydrates, cholesterol, and low saturated fats. Fish also provides high-value protein and a wide range of essential micronutrients such as vitamins, minerals, and polyunsaturated omega-3 fatty acids. Because of the nutritional and health benefits of fish and other seafoods, the demand is always high and annual consumption is increasing globally. For example, the demand for seafood in Australia has steadily increased over the last three decades [119]. In 2009, Australians on average consumed 25 kg of seafood, compared with 18.8 kg in 1995, 17.3 kg in 1985, and 13.6 kg in 1975. The data indicates that overall seafood consumption in Australia has almost tripled over three decades [118]. In parallel with the increasing global demand for seafood, there are growing concerns about the sustainability and management of the fishing industry. Recent studies have only discussed wastage in general terms and suggest that waste could be as large as US$ 50 billion each year due to poor management of seafood resources [120, 121]. A recent article by Costello et al. illustrated how fish waste could be reduced in a sustainable way if appropriate management changes were undertaken. The study highlighted that the less studied fisheries have not been closely monitored or assessed, so there is no data recording the amount of waste being produced [122].

**Figure 3:**
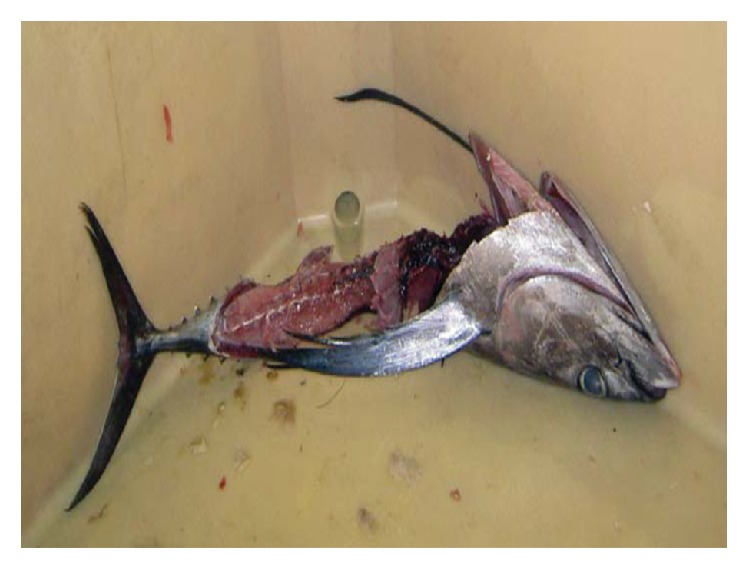
During processing, the fillets are considered usable and the remainder is waste.

Different types and quantities of fish waste are produced throughout the food supply chain, commencing with capture and ending with consumption [123]. Worldwide, around 130 million tonnes of fish waste is produced each year by fisheries and aquaculture. Wastes are produced through by-catch, on-board processing, transport, storage, retailers, and consumers [124]. Fish waste generation begins during wild catching, with by-catch or unintentional catching of marine species being discarded. This problem has been extensively studied and in spite of environmental and business guidelines there is still no effective solution to by-catch waste [125, 126]. It is estimated that globally around 17.9 to 39.5 million tonnes of whole fish is discarded each year by commercial fishing operations [123]. Following capture, processing is the main stage in the food supply chain where most waste occurs. During processing, only the fillets are preserved and the remainder of the fish (up to 66%) is thrown away as seen in Figure 3 [127, 128]. A study by Gavine et al. found that the southeastern Australian seafood industry produced fish waste estimated to be around 20,000 tonnes per year and cost around US$ 150 per tonne to dispose of in landfill sites [129]. In reality, not only does the waste disposal have a significant cost, but also it has a major environmental impact [130].

Interestingly, in the UK, each tonne of cod purchased by a processor costs about £2,000 (US$ 3,129) and around 50% of the cod ends up as processing waste. Regrettably, the waste only generates an income of £40 (US$ 63) as a byproduct and in the worst-case scenario its disposal costs around £60 (US$ 94). Similarly, only around 43% of shellfish and other fish species are suitable for human consumption and the remaining are classified as waste [123]. It has been estimated that in the UK both meat and fish processing were responsible for about 56% of all wastes produced in the food supply chain as shown in Table 6 [36, 93]. A study of a grouping of 14 European countries revealed that fish processing waste could range between 40 and 70% [94]. Thus, it is apparent from these studies that the processing stage is the main contributor to overall waste levels. Research into retail and consumer waste has shown that consumers in the USA are the major contributor to fish waste. Interestingly, the trend in waste by consumers has steadily increased from 16% in 1995 to 31% in 2010 despite having efficient transport and storage facilities [13, 17, 58]. The reasons for the high levels of waste by USA consumers are unknown and need further investigation.

**Figure 4:**
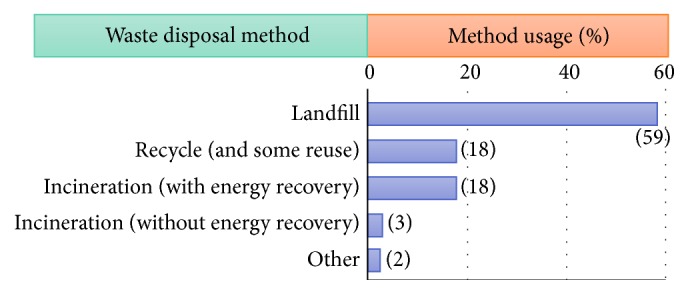
Scottish salmon farming waste disposal routes [131].

The disposal route for seafood waste is not as straightforward as grains and other crop products. This is because the disposal of seafood wastes involves stricter hygiene, safety, and management of environmental hazards during its disposal and in many cases its disposal is regulated by government organisations. For example, in the UK, landfill costs are much higher for seafood waste disposal because the waste is not categorised as “inactive/inert” waste. Furthermore, regulations regarding the burial or burning of seafood waste are restrictive if there are any alternative utilisation pathways available [123]. However, some fish farming businesses are paying higher landfill costs to dispose of fish wastes compared to other methods of disposal. Currently, around 59% of all fish wastes go to landfill and only around 39% are reused or incinerated as seen in Figure 4 [131].

**Figure 5:**
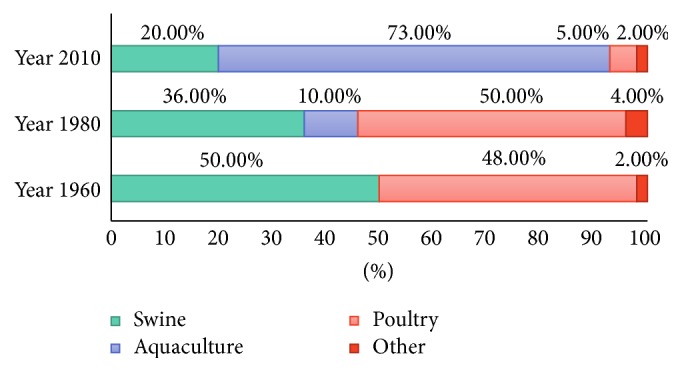
Global usage of fishmeal (adapted from World Bank data) [120].

To reduce the large amount of fish waste produced worldwide, a number of alternative strategies have been developed to add economic value to the wastes. For instance, two different methods, mass transformation and sorting, have been developed to improve the economic value of fish wastes [124]. Mass transformation involves the conversion of fish waste into a single product. Typical examples of transformed fish waste include fishmeal, fish oil, fertilisers, and hydrolysates such as protein hydrolysate. Alternatively, sorting involves utilising various fish body parts such as bones, guts, and fins separately to enhance their economic value. For example, sorting enables the production of specialised products such as liver oil, gelatine, omega-3, protein containing sports food and drinks, calcium, cosmetics, and pharmaceuticals [132]. Wider acceptance and adoption of both methods could lead to significant reductions in wastes going to landfill and reduce the damaging impact of fish wastes on the environment. For example, converting fish wastes into fishmeal has been steadily increasing in recent years with many countries converting their fish wastes using cost-effective reprocessing technologies [118, 133]. In spite of the reprocessing costs associated with converting fish waste into fishmeal, fishmeal's value as a feedstock for aquaculture has offset the reprocessing costs. For example, in Japan, 90% of the ingredients used in fishmeal are derived from fish wastes [134]. Currently, there are only around ten major countries converting fish waste into fishmeal products, that is, Canada, Chile, Denmark, Iceland, Japan, Mexico, Norway, Russian Federation, Thailand, and the USA. However, these countries on average are only using around 25% of their fish wastes to produce fishmeal products [133]. Importantly, fishmeal contains essential amino acids and as a result it is currently the most widely used protein ingredient in aquaculture feeds [135]. Thus, fishmeal usage over a 50-year period (1960 to 2010) reveals its increased use in aquaculture, while its use in both swine and poultry feeds has declined as seen in Figure 5 [136]. One of the contributing factors for this trend was the ban imposed by the European Economic Commission on the use of animal byproducts being used in animal feeds. And similar regulations in the USA have also contributed to the increased usage of fishmeal in aquaculture [137].

In spite of fishmeal being used globally, there has only been limited use of other fish waste byproducts. Fish wastes can also be processed to produce oil, silage, fertiliser, composting matter, and fish protein concentrates [138]. Furthermore, fish wastes are also a rich source of chitin, chitosan, carotenoid pigments, and enzymes that can be used in cosmetics and pharmaceuticals [139]. But, to date, very little has been done to fully develop and commercialise these types of products [123]. However, it should be noted that fish waste processing can be a difficult business in many countries due to problematic issues such as hygiene, safety, and environmental hazards. In addition, the most important factor that any business needs to consider is the economic viability of fish waste processing [140, 141]. For example, large volumes of both solid and liquid wastes are produced after processing Nile perch from Lake Victoria in East Africa. Annually, around 36,000 tonnes of solid waste and approximately 1,838,000 m^3^ of produced wastewater containing valuable nutrients are discharged [142]. An investigation of the wastewater revealed that it contained 6,160 mg/L of lipids and 2,000 mg/L of protein [143]. This rich source of lipids and proteins has the potential to produce value-added products through bioconversion. However, current fish waste management in East Africa was found to be inefficient and nonprofitable and was unable to take advantage of the rich source of lipids and proteins present in the wastewater, thus highlighting the need for efficient waste utilisation and waste reduction strategies that can provide viable and profitable options for fish waste processing [142].

A number of aquaculture based industrial studies have examined various types of methods for dealing with seafood waste and its utilisation in Australia [136, 144, 145]. For example, fish wastes are a rich source of essential fatty acids and fish skin-and-bone parts are suitable mineral supplements in fish diets [146]. However, further studies are needed to fully investigate large-scale profitable fish waste processing. On the whole, fish waste processing and utilisation have steadily increased over the years, but several issues restrict its full-scale operation. In particular, environmental issues are major factors preventing large-scale development since fish processing plants can be significant polluters. Obviously, there are good economic and environmental reasons to process fish waste and produce value-added products. But further work is needed to develop effective and efficient methods of processing fish wastes at an economically viable industrial scale with as little environmental impact as possible.

### 3.2. Aquatic Plant Based Wastes in the Food Supply Chain

**Figure 6:**
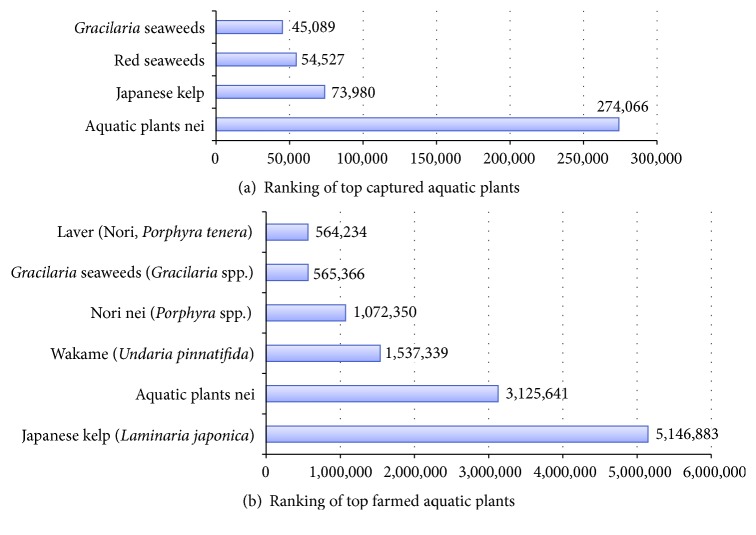
Captured and farmed aquatic plant food species in 2010 (data in tonnes) [118, 156].

It is interesting to note that the literature in the field often overlooks aquatic plant food wastes. Aquatic plant foods such as algae have been used for both human and animal nutrition for thousands of years. The earliest writings of the ancient Greeks recorded in the* Bellum africanum*, written around 45 B.C., describe the Greeks collecting seaweed from local shorelines and feeding it to their cattle [147]. Many aquatic plants are very rich in protein and are a highly nutritional food that can offer many beneficial advantages as a food supplement as well as having significant medicinal properties [148–150]. In the search for sources of natural antioxidants, algae and microalgae have been suggested as potentially rich sources. Both algae and microalgae are widely known and consumed in many countries for their advantageous health benefits. In particular, many algae and microalgae are rich sources of polyunsaturated fatty acids that have the potential to reduce the incidence of cardiovascular diseases [151, 152]. In Asian countries like China, Japan, and Korea, the production and consumption of edible aquatic plants had a long tradition. This long-standing tradition has resulted in the widespread incorporation of aquatic plants into the global food supply [153–155]. Rather than just relying on marine capture, currently over 95.5% of the total global production of aquatic plants is supplied by aquaculture [156]. This equates to around 0.44 million tonnes of marine capture and about 12 million tonnes being produced by aquaculture in 2010 as seen in Figure 6.

Studies have shown that the majority of aquaculture production, around 9 million tonnes, was destined for human consumption. Phycocolloids were extracted from the remaining aquatic plants to be used as nutritional supplements in various forms of farm animal and aquaculture feedstock [156, 157]. To date, there has been very little data reported in the literature and wastes levels produced by aquatic plant food industries remain relatively unknown. Likewise, the management of wastes produced during processing remains largely unknown. Therefore, there is a current need to undertake research into aquatic plant food supply chain to determine the current amount of waste, level of utilisation, and management protocols in use.

However, in recent years, research has focused on using microalgae in the production of biodiesel. Microalgae have two major advantages over land based crops. The first is the high growth rate and the second is the high oil content. For example, microalga typically doubles its biomass every 24 hours under normal growing conditions, while the oil content of microalgae can range from 15 to 75% (dry weight) and annually can produce oil from 58,700 up to around 136,900 litres per hectare [158, 159]. Currently, biodiesel production depends on crops such as soybean, rapeseed, canola, sunflower, corn, palm kernels, animal fat, and oils [160]. The biggest hurdle preventing the full-scale production of biodiesel from these crops is land availability [161]. Since the land area needed by microalgae is small compared to oil producing crops, there has been considerable interest in exploring the use of microalgae as an alternative feedstock for the production of biodiesel. The disadvantage of using microalgae for producing biodiesel is the high cost of production and separation that is needed to remove microalgal biomass from the growing media [159, 162]. Another challenge associated with microalgae production in open ponds is contamination from a wide range of naturally occurring algae and bacteria [160]. Similarly, microalgae have also been considered for producing bioethanol. But similar issues encountered for biodiesel production are also prevalent for bioethanol production such as algal biomass separation and contamination [163, 164]. Interestingly, if aquatic plant food wastes proved suitable, they could also be evaluated as a possible feedstock for the production bioenergy products. But this possible application of aquatic plant food wastes needs to be investigated.

## 4. Discussion

**Figure 7:**
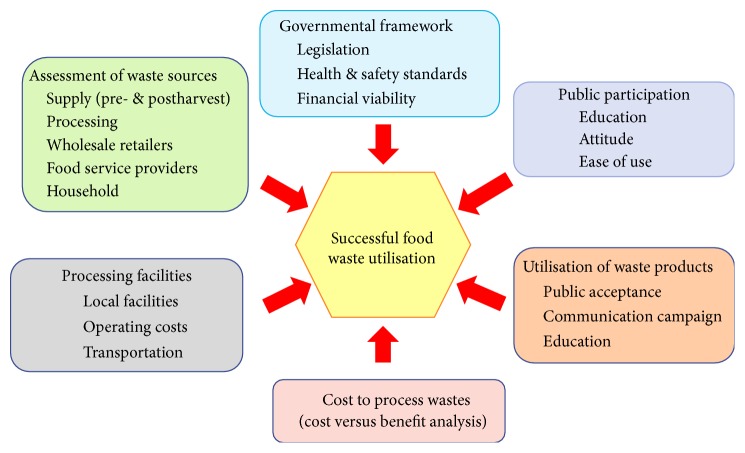
Important factors that need to be considered for successful utilisation of a food waste based product.

At present, there is very little information in the literature discussing the industrial scale utilisation of food wastes at the local, national, or international level. For example, fruit and vegetable wastes have been extensively studied and reported in the literature. However, food wastes produced and their utilisation in aquaculture, livestock, poultry, and dairy industries are rarely reported and need further research. Much of the currently available food waste data lacks sufficient details and there is even less information discussing waste utilisation in the respective food supply chains. This information is needed before any economic modelling can be done to determine the feasibility of new products and waste transforming facilities needed to produce a commercially successful business outcome. The first step in developing a successful waste utilisation strategy is to assess the type and magnitude of waste [165, 166]. Once waste levels and their location in the food supply chain are known, it is now possible to start developing an effective waste management plan. In developing an effective plan, several important factors need to be considered before successful waste utilisation can be achieved as seen in Figure 7. Ultimately, the main barrier to developing any waste management plan that produces a new product from food waste needs to take into account several strategic factors, for example, new market opportunities, market trends, current market developments, and producing a product that is competitive in the marketplace [167]. Furthermore, each stage of product development needs to be carefully considered. In the case of manufacturing, a company will need to consider commercial opportunities based on a well-thought-out growth strategy, especially if innovation is a key factor of the product. For packaging and distribution, the product range and associated services will also need to be carefully considered with the view of preventing competitor copying and safeguards to maintain market share. From a governmental perspective, policies may need to be formulated that promote sustainable patterns of consumption and sustainable community lifestyles, foster new job creation strategies, and enhance the economy. For consumers, the combination of diversity, choice, and expectation of high quality produce is a very important issue in their selection process. In summary, any new product produced from a food waste utilisation process that enters the marketplace will need to be both economically and ecologically sustainable. However, at the end of the day, it is consumer acceptance of the new product that is the deciding factor [168].

A recent study by Kummu et al. found that the preferred option for food waste utilisation was to use wastes generated from agriculture and consumers. From a global perspective, their study suggested that 47% of agricultural food wastes and over 86% of consumer wastes could be effectively utilised. The study also found that the biggest improvements in food waste management would occur where the demand for additional food was the least [169]. Therefore, to effectively manage food waste, there needs to be awareness of the benefits of postharvest waste utilisation by farmers, food processors, and government agencies. This awareness is needed so that food waste management capacity can be built up and ultimately lead to improvements in converting wastes into value-added products [54]. Importantly, it is also necessary to fully understand the size of the problem so that there are opportunities to improve food security and reduce poverty using effective waste utilisation strategies. In addition, the reduction of food wastes by effective waste management also reduces environmental degradation and improves economic sustainability of the food supply chain. However, the most important factors that will contribute to the success of any food waste utilisation strategy are its acceptance by the community at large.

Whenever food waste utilisation is debated, it is generally discussed in terms of processing methods, but actual food supply chain losses and their true impacts are often undervalued and underreported [170]. Undervaluing and underreporting are commonly referred to as the “*hidden costs*” of food waste management. Exploring these “*hidden costs*” usually acts as a catalyst for determining the scale of the waste problem, since businesses will only become aware of the problem when it impacts their bottom line. Generally, food related businesses often resolve their waste management problems by keeping their profitability levels high. They usually achieve this goal by reducing energy consumption, reducing raw material usage, and improving recycling activities [171]. Furthermore, businesses will investigate the merits of managing wastes in terms of recovery and value adding as opposed to the cost of disposal [172]. In fact, the disposal cost will have a direct impact on whether a business will go down the recovery and value-adding option or take the waste disposal route [18]. Therefore, food waste management options will often involve a cost versus benefit analysis that ultimately determines businesses profitability. However, because of business confidentiality reasons, food waste management costs are normally not reported. This often leads to partial and unproven estimates of the impact of food waste and makes assessments of waste management strategies difficult [173]. For instance, in many developed countries, the main driver for waste management strategies is government legislation relating to safety, handling of hazardous waste materials, and the environmental impact of the businesses operational practices. In developing countries, factors such as food type, processing facilities, storage facilities, transport, and even climatic conditions are the principal drivers in food waste management strategies [174]. For example, the drivers for fish, meat, and poultry waste utilisation are health safety and hygiene risks associated with processing the wastes, whereas the economic drivers for fruit and vegetable waste management include microbial spoilage, costs of drying, storage, and shipment of byproducts [175]. Furthermore, these drivers become even more demanding if the food wastes are to be converted into high quality functional compounds [176]. Therefore, waste processing strategies must be optimised to promote production efficiency and cost-effectiveness so that the final products are competitive in the marketplace [177]. Consequently, a cost-and-benefit analysis is of paramount importance before any business adopts a food waste utilisation and management strategy.

The most important factor that needs to be carefully considered when planning to adopt a food waste utilisation strategy that aims to produce value-added products is the consumer. Experience has shown that consumers are often reluctant to accept new products, even when they can see its benefits. Many studies have shown that consumers do not compromise on product quality or performance. This is why consumer behaviour or habit needs to be fully understood when developing and marketing any new product. For example, surveys have consistently shown that consumers are very concerned about the environment and whether new products are ecofriendly [178–180], with consumer queries often focusing on whether environmental guidelines were followed during product manufacture. In Australia, around 62% of all consumer queries involve issues concerning environmental impact [181]. A similar study in Sweden found that customers ranked product taste first, while environmental impact was ranked second [182]. The results of both studies clearly indicate the importance of environmental issues to consumers and how this translates into their purchasing behaviour. These results also emphasise the importance of educating consumers on the ecofriendly nature of products processed from food wastes and their positive impact on reducing environmental degradation. Education is particularly important since most consumers are only aware of industrial pollution and wildlife conservation [183]. In fact, consumer knowledge relating to the production and distribution of food they purchase and its environmental impact is poor. Thus, consumers need product information so that they can make informed decisions and make ecofriendly based choices when selecting products [24]. Providing information in the form of fact sheets at the point of sale or by environmental indicator labelling on product packaging would assist consumers in making informed decisions. In recent years, consumers have become more aware of increasing costs of gas, electricity, and petrol prices. Accordingly, consumers have been encouraged to reduce their home energy consumption using a number of strategies aimed at improving domestic energy efficiency. Unfortunately, no similar strategies have been aimed at raising the awareness of food waste utilisation. In fact, very few strategies have highlighted the negative environmental impact of dumping food wastes in landfill sites and subsequent greenhouse gas emissions. Thus, consumer education and acceptance of value-added products derived from food wastes will ultimately determine the success of any food waste utilisation and management strategy. Through education, consumers will see the value of these waste derived products and their positive environmental impact. This will ultimately influence consumer behaviour and promote purchasing patterns towards food waste derived value-added products.

## 5. Conclusion

Today, there is a general absence of detailed information and understanding of the extent of food wastage at different stages of the food value chain from farm to fork. The scale of food waste throughout the food supply chain is complex and can have a significant impact on a number of different fields such as agriculture, food security, economics, waste utilisation and management, environmental conservation, and human health. To resolve food waste problems and promote food waste utilisation strategies in any country will require effective communication and cooperation between all stakeholders. There are a number of hurdles preventing the conversion of food waste to value-added products. These hurdles include developing effective cost/benefit food waste utilisation strategies, developing efficient ecofriendly reprocessing technologies, reducing environmental degradation, and public acceptance. Globally, a number of countries are tackling the problems associated with increasing food waste and food waste utilisation and management. For example, several European countries are promoting utilisation and management strategies such as bioenergy production and regulating landfill costs to discourage waste generation. The key to successful food waste utilisation and management is to develop appropriate ecofriendly reprocessing technologies that can convert all the valuable components present in the waste into valuable products and reduce the amount of waste going to landfill. However, there are many challenges that must be overcome to achieve this goal. Consumer awareness and education is one such challenge. Without consumer acceptance of food waste reduction approaches, no sustainable ecofriendly food waste utilisation and management strategy can succeed. The present work has also identified the need for more detailed studies identifying where, why, and how much food waste is produced between farm and fork.

## Figures and Tables

**Table 1:** Representative global examples of food loss (waste) [11].

Food loss (waste)	Reference
In the USA alone, annual food production consumes about 120 cubic kilometres of irrigation water. People throw away 30 percent of this food, which corresponds to 40 billion litres of water.	[184]
United Kingdom households waste an estimated 6.7 million MT of food every year, around one-third of the 21.7 million MT purchased. This means that approximately 32 percent of all food purchased per year is not eaten. Most of this (5.9 million tonnes or 88 percent) is currently collected by local authorities. Most of the food waste (4.1 million MT or 61 percent) is avoidable and could have been eaten if it had been better managed.	[185]
The amount of food lost or wasted every year is equivalent to more than half of the world's annual cereals crop (2.3 billion MT in 2009/2010). Only an estimated 43 percent of the cereal produced is available for human consumption, as a result of harvest and postharvest distribution losses and use of cereal for animal feed.	[186]
The water applied globally for irrigation to grow food that is wasted would meet the domestic needs of 9 billion people.	[187]
Annual food losses and waste are estimated at about 30 percent for cereals, 40 to 50 percent for root crops, 30 percent for fish, and 20 percent for oilseeds and meat.	[188]
On a global scale, just 43 percent of the fruits and vegetables produced are consumed and the remaining 57 percent are wasted.	[189]
Food waste accounts for roughly US$ 680 billion in industrialised countries and US$ 310 billion in developing countries.	[190]
Consumers in rich countries waste about 222 million MT of food every year, which is nearly equivalent to the entire net food production of 230 million MT of sub-Saharan Africa.	[185, 191]
Roughly one-third of food is lost or wasted. That translates into 1.3 billion MT each year, worth nearly one trillion US dollars, and is the equivalent of 6 to 10 percent of human-generated greenhouse gas emissions.	[192]
Food spoilage and waste account for annual losses of US$ 310 billion in developing countries, where nearly 65 percent of loss occurs at the production, processing, and postharvest stages.	[192]
In sub-Saharan Africa, up to 150 kg of the food produced per person is lost each year; depending on the crop, 15–35 percent of food harvested may be lost before it leaves the field.	[192]

**Table 2:** Food wastes produced in the food supply chain as reported in the literature.

Food supply chain stage	Cause of food waste	Reference
Production and harvest	Crops left in ground; not meeting quality standard	[16, 17, 193]
	Overproduction to maintain supply	[17]
	No demand right at that time of harvest	[194]
	Wrong forecast/withdrawal of demand from retailers	[195]
	Fall of crops and livestock prices	[194]
	Failure to meet quality standards	[4]
	Lack of coordination within the supply chain	[6]
Storage	Pests/diseases attacking/destroying crops	[173]
	Lack of storage facilities	[6, 17]
	Livestock death and unsuitability for slaughter	[87]
	Lack of suitable refrigeration	[194]
	Shortened shelf-life promoting more food waste	[196]
Processing and handling	Trimming (shape, size) for attractive visual appearance	[6, 35]
	Crops nonedible or unsuitable for canning, livestock trimming during slaughtering or fish during canning/smoking, filleting	[6, 17]
	Dairy products during pasteurization and processing to milk based products	[87]
Transport and distribution	Excessive transportation	[197]
	Longer periods of inactivity and complex and expensive movements resulting in product damage	[197–199]
Retail	Products sorting to meet supermarket quality standard	[6]
	Products not donated due to safety standard	[194]
	Expiry of products such as meat and milk before being purchased	[200]
	Maintaining high standard and consumer attraction	[201]
	Packaging size not suitable for buyers	[87]
	Product/packaging damage and being not attractive to consumers	[202]
	Excessive awareness of “due date,” “use by” date, “expiry date”	[194, 203]
Consumer	Buying behaviour and purchasing pattern	[15, 200]
	Family size, income, age, job pattern	[19, 204]
	Excessive buying without need	[58, 197, 201, 203]
	Misunderstanding/lack of knowledge about labelling	[173, 197, 205]
	Product purchased but not processed/cooked	[36]
	Surviving more on takeaway food while fridge is still full/no time to cook	[200]
	Cooked product not tasty enough to eat	[206, 207]
	Product expired and produce that is wilted/bruised/moulded and is thrown away	[36, 197]

**Table 3:** Amount/percent/value of fruit and vegetable waste in the world food supply chains.

World zone	Loss amount	Stage of waste	Calculation method	Reference
UK	36%	Household		[36]
Switzerland	47% (veg. only)	Production, postharvest handling, processing	Share of losses calculated and estimated in percentage	[8]
	11% (veg. only)	Retail		
	40% (veg. only)	Household		
Germany	43%	Household	Share of total footprint created	[208]
UK	8%	Food processing industries	Percentage	[36, 93]
14 European countries^*∗*^	5–30%	Food processing industries	Percentage of total share	[94]
Sweden	4.3%	Retail	Percentage share of total delivered products in the retail stores	[209]
China	15% 10%	Storage Distribution	Average loss in China calculated from data published by several researchers	[210]
China	25–35%	Storage	Percentage loss in 2011	[211]
Australia	US$ 810	Consumer waste	Average annual waste value per person	[19]
Africa	53% (incl. root and tuber)	Total supply chain	Percentage of total share	[62]
Sub-Saharan African	10%	Production	Percentage (by mass)	[6]
	9%	Postharvest handling and storage		
	25%	Processing and packaging		
	17%	Distribution		
	5%	Consumption		
South America	6.28%	Wholesale		[212]
Brazil	8.76%	Retail		
North America	48.7% (fresh and processed)	Supply chain	Total weight in lb. (pound) (data collected by USDA in 1995)	[59]
USA	18% 33%	Retail Consumer	Estimated total value of food loss in 2008	[13]
Waterloo, Ontario, Canada	16%	Household	Average of reported food wastage percentages for online survey participants	[213]

^*∗*^14 European countries: Portugal, Spain, France, Netherlands, Belgium, United Kingdom, Sweden, Finland, Denmark, Germany, Italy, Poland, Hungary, and Greece.

**Table 4:** Analysis of retail and consumer waste increase/decrease in the USA based on USDA data from [17, 58, 60].

Commodity		Supply/population (S/P)^*∗*^			Supply/waste (S/W)			Production% increase/decrease		
		1995	2008	2010	1995	2008	2010	1995	2008	2010
Grains	R	17.13	19.55	19.50	0.02	0.12	0.12	2	+10	+10
	C				0.30	0.18	0.19	30	−12	−11
Fruits	R	18.15	22.01	20.76	0.02	0.09	0.09	2	+7	+7
	C				0.22	0.14	0.19	23	−9	−4
Vegetables	R	23.69	36.96	27.09	0.02	0.06	0.08	2	+4	+6
	C				0.24	0.15	0.22	24	−9	−2
Dairy products	R	28.64	27.48	26.80	0.02	0.11	0.11	2	+9	+9
	C				0.30	0.17	0.19	30	−13	−10
Meat/poultry	R	17.82	27.13	17.31	0.01	0.03	0.04	1	+3	+3
	C				0.15	0.23	0.21	15	+8	+6
Fish	R	1.50	1.59	1.55	0.01	0.08	0.08	1	+7	+7
	C				0.15	0.25	0.31	15	+10	+16
Eggs	R	2.97	2.89	3.16	0.02	0.1	0.07	2	+8	+5
	C				0.29	0.15	0.21	29	−14	−8
Nut products	R	0.71	1.04	1.13	0.01	0.06	0.06	1	+5	+5
	C				0.15	0.09	0.09	15	−6	−6

R: retail waste; C: consumer waste.

^*∗*^Population in 1995 = 266.3 million; in 2008 = 304.06 million; in 2010 = 309.75 million (source: ERS).

**Table 5:** Amount/percent/value of grain waste in selected world food supply chains.

World zone	Loss amount	Stage of waste	Calculation method	Reference
China	4–6%	Postharvest handling	Average loss in China calculated from data published by several researchers	[211]
	5.7–8.6%	Storage		
	2.2–3.3%	Processing		
	1–1.5%	Distribution		
China	7–10%	Storage	Percentage loss in 2011	[211]
Australia	US$ 435 (grain products)	Consumer	Average annual waste value per person	[19]
Switzerland	62% (grain products)	Production, postharvest handling, processing	Share of losses calculated and estimated in percentage	[8]
	4% (grain products)	Retail		
	32% (grain products)	Household		
Africa	26%	Total supply chain	Percentage of total share	[62]
Sub-Saharan Africa	6%	Production	Percentage (by mass)	[6]
	8%	Postharvest handling and storage		
	3.5%	Processing and packaging		
	2%	Distribution		
	1%	Consumption		
North America	32%	Supply chain	Total weight in lb. (pound) (data collected by USDA in 1995)	[59]
USA	12% 18%	Retail Consumer	Estimated total value of food loss in 2008	[13]

**Table 6:** Amount/percent/value of meat and poultry waste in the world food supply chains.

World zone	Loss amount	Stage of waste	Calculation method	Reference
UK	7% (meat & fish)	Household		[36]
UK	56% (meat & fish)	Processing industries	Percentage	[36, 93]
14 European countries^*∗*^	35–42%	Processing industries	Percentage of total share	[94]
China	1.4–2.1%	Postharvest handling	Average loss in China calculated from data published by several researchers	[210]
	2.5–3.7%	Storage		
	1.1%	Processing		
	3%	Distribution		
Australia	US$ 626 (meat & fish)	Consumer	Average annual waste value per person	[19]
Africa	7%	Total supply chain	Percentage of total share	[62]
Sub-Saharan Africa	15%	Production	Percentage (by mass)	[6]
	0.7%	Postharvest handling and storage		
	5%	Processing and packaging		
	7%	Distribution		
	2%	Consumption		
North America	16% (including fish)	Supply chain	Total weight in lb. (pound) (data collected by USDA in 1995)	[59]
USA	5% 35%	Retail Consumer	Estimated total value of food loss in 2008	[13]
Waterloo, Ontario, Canada	6% (including seafood and eggs)	Household	Average of reported food wastage percentages for online survey participants	[213]

^*∗*^14 European countries: Portugal, Spain, France, Netherlands, Belgium, United Kingdom, Sweden, Finland, Denmark, Germany, Italy, Poland, Hungary, and Greece.

**Table 7:** Amount/percent/value of eggs, milk, and dairy waste in the world food supply chains.

World zone	Loss amount	Stage of waste	Calculation method	Reference
*Eggs*				
UK	7% (incl. dairy)	Household		[36]
Switzerland	18%	Production, postharvest handling, processing	Share of losses calculated and estimated in percentage	[8]
	9%	Retail		
	64%	Household		
North America	31.4%	Supply chain	Total weight in lb. (pound) (data collected by USDA in 1995)	[59]
USA	9% 14%	Retail Consumer	Estimated total value of food loss in 2008	[13]
*Milk and dairy*				
UK	12%	Food processing industries	Percentage	[36]
14 European countries^*∗*^	43%–48%	Food processing industries	Percentage of total share	[94]
Australia	US$ 405	Consumer	Average annual waste value per person	[19]
Africa	8%	Total supply chain	Percentage of total share	[62]
Sub-Saharan Africa	6	Production	Percentage (by mass)	[6]
	11	Postharvest handling and storage		
	0.1	Processing and packaging		
	10	Distribution		
	0.1	Consumption		
North America	32.0%	Supply chain	Total weight in lb. (pound) (data collected by USDA in 1995)	[59]
USA	9%	Retail	Estimated total value of food loss in 2008	[13]

^*∗*^14 European countries: Portugal, Spain, France, Netherlands, Belgium, United Kingdom, Sweden, Finland, Denmark, Germany, Italy, Poland, Hungary, and Greece.
